# The Effect of Adiponectin on Osteonectin Gene Expression by Oxidized Low Density Lipoprotein-Treated Vascular Smooth Muscle Cells

**Published:** 2015

**Authors:** Sara Niknam, Keihan Ghatreh-Samani, Effat Farrokhi

**Affiliations:** 1 *1.Clinical Biochemistry Research Center, Shahrekord University of Medical Sciences, Shahrekord, Iran.*; 2 *2.Cellular and Molecular Research Center, Shahrekord University of Medical Sciences, Shahrekord, Iran.*

**Keywords:** Vascular smooth muscle cells, oxidized low density lipoprotein, adiponectin, osteonectin

## Abstract

Osteonectin is a bone- associated protein involved in vascular calcification. Adiponectin may protect against cardiovascular disease but possible effects on vascular calcification have been poorly studied. The aim of this study was to investigate the modulatory effect of adiponectin on oxidized low density lipoprotein (oxLDL)- induced expression of osteonectin in human aorta vascular smooth muscle cells (HA/VSMCs). HA/VSMCs were cultured in F12K media and then treated with oxLDL (100 µg/mL) in the presence or absence of adoponectin (5 µg/mL) for 24 and 48 hours. mRNA expression and protein level of osteonectin were determined by quantitative real-time PCR and western blot analysis, respectively. After exposure to oxLDL, osteonectin expression increased 1.62 ± 0.23- and 6.62 ± 0.48-fold after 24 and 48 hours respectively compared to the control. Adiponectin increased oxLDL- induced osteonectin expression in a time-dependent manner after 24 and 48 hours (3.24 ± 0.39- and 24.93 ± 2.15-fold, respectively). Western blotting confirmed that osteonectin protein was upregulated by adiponectin.Our data suggest that OxLDL might cause the increase of osteonectin expression both at mRNA and protein level. This upregulation is intensified by adiponectin.

Atherosclerosis is a disorder characterized by the presence of atherosclerotic plaques in the arterial intima that leads to luminal narrowing (1, 2). Calcification is a common event that occurs in the early phase of arteriosclerosis (3, 4) which is predisposing to coronary artery damage after angioplasty (5). Calcification reduces the blood walls elasticity that can lead to other cardiovascular problems (6). Oxidized low density lipoprotein (OxLDL) has been postulated to play an important role in foam cells formation and its accumula-tion in the vascular wall which stimulates the development of atherosclerosis and vascular calcification (7).

Osteonectin, also known as secreted protein acidic and rich in cysteine (SPARC) is an extracellular matrix protein expressed in active remodeling in the skeleton and other tissues (8).

As with other bone-related proteins, osteonectin is expressed in the arterial wall during atherosclerosis progress specifically during calcification of the atherosclerotic plaque (9-11).

Adiponectin is one of the adipocytokines released only from adipose tissue, which protects from the development and progression of athero-sclerosis via anti- inflammatory effects. Decreased plasma adiponectin concentrations were reported in patients with coronary artery disease (12). Experi-mental studies have shown that adiponectin has potential antiatherogenic characteristics (12, 13).

Several experimental findings suggest that adiponectin may protect against cardiovascular inflammation (14, 15), but the possible effects of adiponectin on vascular calcification have not been fully investigated. Adiponectin probably reduces calcification process and therefore can be effective on reducing the risk of atherosclerosis (16-19). The aim of the present study was to investigate the effect of adiponectin on osteonectin expression in vascular smooth muscle cells (VSMCs) treated with oxLDL.

## Materials and methods

**Cell culture**

In this experimental study, human aorta vascular smooth muscle cells (HA/VSMCs) were cultured in F12K media containing 0.05 mg/mL ascorbic acid, 0.01 mg/mL insulin, 0.01 mg/mL transferrin, 10 ng/mL sodium selenite, 0.03 mg/mL endothelial cell growth supplement, 10% FBS (final concentration), 10 mM 4-(2-hydroxyethyl) pipe-razine-1-ethanesulfonic acid solution (HEPES), 10 mM N-tris (hydroxymethyl) methyl-2-aminoethan-esulfonic acid (TES), 100 U/mL penicillin, 100µg /mL streptomycin, and 0.01% amphotericin B. Cells were incubated in the humidified incubator at 37 °C with 5% CO2. Cells were used at passa-ges 3-7.

Cells were seeded in a 12- well cluster plates at a density of 10000 cells per well. When the cells achieved approximately 80% confluence, they were treated with either 100 µg/mL oxLDL (Biomedical Technologies Stoughton, MA, USA) or a combi-nation of oxLDL and 5 µg/ mL adiponectin (Bio-vendor, Heidelberg, Germany) in the presence of 10 mM β-glycerophosphate. Cells without any treat-ment were used as control.

**RNA isolation and cDNA synthesis**

Total RNA was extracted from the cells using Biozol RNA extraction reagent (Bioflux, Japan) after 24 and 48 hours. The RNA was quantified using a nanodrop spectrophotometer (Thermo Fisher Scientific, USA) at 260 nm. The cDNA was synthesized from 0.3 µg total RNA using the Revert Aid First Standard cDNA Synthesis kit (Thermo Fisher Scientific ,Waltham, USA).

**Real-time polymerase chain reaction (PCR)**

Real- time PCR was performed using Rotor-Gene 3000 real-time DNA amplification system (Corbett Research, Australia) and SYBR green method. Primers used for real-time PCR are listed in Table 1. Experiments were performed in triplicate. Each multiplex reaction mix (25 μl) contained 12.5 μl of SYBR Green PCR Master Mix (Qiagen, Valencia, USA), 0.3 µl of each primer (10 µM), 3 µl cDNA (20 ng), and 8.9 µl nuclease-free water. The amplification was carried out under the following conditions: initial denaturing at 94 °C for 5 minutes, then 40 cycles of 95 °C for 15 seconds, 59 °C for 20 seconds and 72 °C for 30 seconds. Quantitation of data was performed using the comparative C_T_ (ΔΔC_T_) method, using glyceralde-hyde 3-phosphate dehydrogenase gene expression as an endogenous reference.

**Western blot**

Cells were washed twice with cold phosphate buffered saline (PBS) and were lysed in ice-cold 2X radio- immune precipitation assay (RIPA) buffer. The homogenate was incubated in lysis buffer for 30 minutes and then centrifuged at 12000 rpm for 10 minutes. The supernatant was used as total cell lysate. Protein concentration was measured spectro-photometrically by Nanodrop at 280 nm and equal amounts of protein from each sample were subjected to blotting. Protein lysate was mixed with SDS loading buffer (Tris-HCl 0.125 M, SDS 4%, Glycine 20%, 2-Mercaptoethanol 10%), followed by boiling for 5 min, and separated by 12% sodium dodecyl sulfate polyacrylamide gel electrophoresis. The separated proteins were then transferred to a polyvinylidene difluoride membrane (PVDF) in tris-glycine buffer for 2 hours at 120 V.

**Table 1:** Primer sequences and amplicon length

Genes	Primer sequences (5'–3')	Amplicon length (bp)	Gene bank reference sequence
Osteonectin	Forward: TCTTCCCTGTACACTGGCAGTTC Reverse: AGCTCGGTGTGGGAGAGGTA	73	NM-003118.3
GAPDH*	Forward: ACACCCACTCCTCCACCTTTG Reverse: TCCACCACCCTGTTGCTGTAG	112	NM-002046.5

* Glyceraldehyde 3-phosphate dehydrogenase

**Fig. 1:**
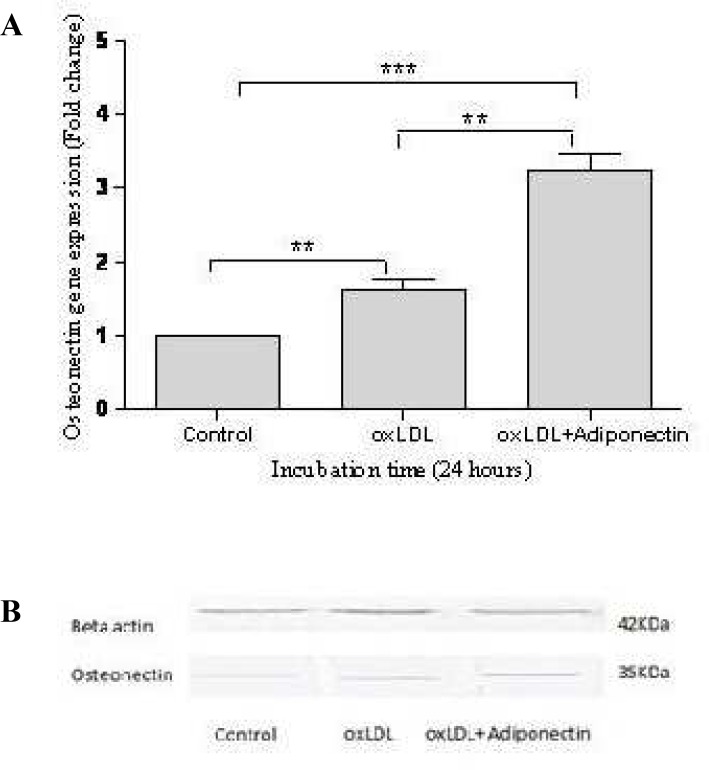
The effect of oxLDL and adiponectin on osteonectin gene expression in HA/VSMCs after 24 hours. (A) Osteonectin mRNA expression against control after treatment with oxLDL alone or together with adiponectin using real time PCR. Data have been expressed as means ± SEM for the three experiments. P values are indicated above the bars: ^** ^P < 0.05, ^*** ^P < 0.01. (B) Western blot analysis showing osteonectin protein level after 24 hours of treatment with oxLDL alone or together with adiponectin. Beta-actin (42 kDa) was used as an internal control to standardize the protein loading in western blotting

**Fig. 2:**
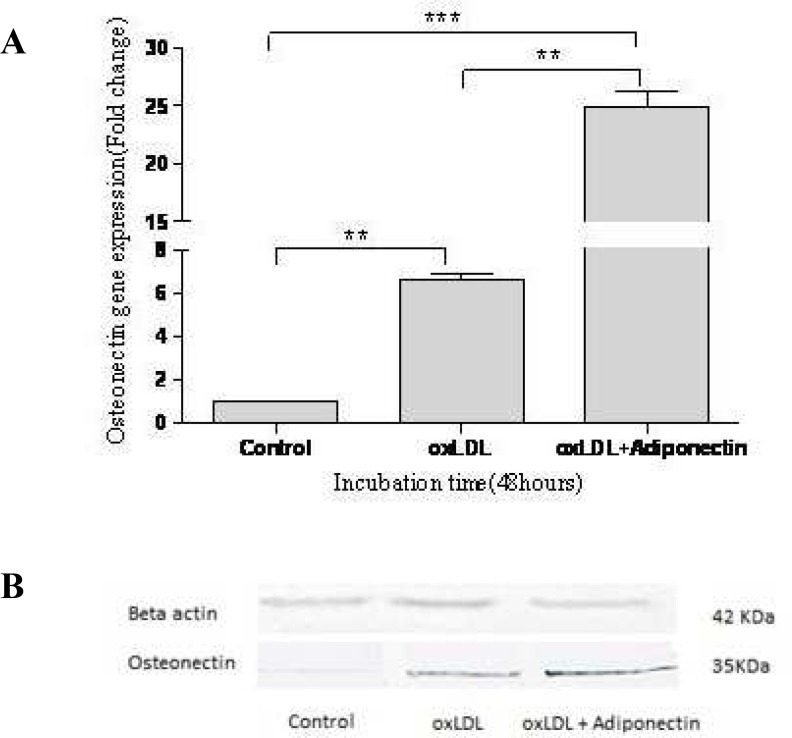
The effect of oxLDL and adiponectin on osteonectin gene expression in HA/VSMCs after 48 hours. (A) Osteonectin mRNA expression against control after treatment with oxLDL alone or together with adiponectin using real time PCR. Data have been expressed as means ± SEM for the three experiments. P values are indicated above the bars: ^**^P < 0.05, ^***^P < 0.01. (B) Western blot analysis showing osteonectin protein level after 48 hours of treatment with oxLDL alone or together with adiponectin. Beta-actin (42 kDa) was used as an internal control to standardize the protein loading in western blotting

The membrane was blocked by 5% nonfat dry milk in tris-buffered saline, 0.1% tween-20 (TSBT) overnight at 4 °C temperature. Then, the membrane was incubated in TSBT containing 5 μg/ mL rabbit polyclonal anti osteonectin antibody (Abcam, Cambridge, UK) for 2 hours at room temperature. After washing, the membrane was incubated with horseradish peroxidase conjugated goat anti- rabbit IgG (Abcam, Cambridge, UK) diluted 1:10000 for 90 minutes at room temperature. Finally, the color was developed with the addition of 3,3',5,5'-tetra-methylbenzidine membrane peroxidase substrate (Roche, Mannheim, Germany). The color reaction was stopped by washing the membranes with distilled water. Cell lysates were detected on a separate membrane with beta actin as a loading control.

**Statistical analyzes**

All experiments were done in triplicate. Statistical analyzes were done using nonparametric Kruskal-Wallis test. Pairwise comparisons between groups were performed by Mann-Whitney test. All statistical analyzes were performed with Graph Pad Prism5 software. All data were presented as mean±SEM and P <0.05 was considered as the level of significance.

## Results

First, we examined the effect of oxLDL on osteonectin gene expression in VSMCs. Our results showed that oxLDL increased osteonectin expression 1.62 ± 0.23- and 6.62 ± 0.48-fold after 24 and 48 hours, respectively compared with the control group (P=0.01) (Figures 1A, 2A). Then, we examined the effect of adiponectin on osteonectin expression in oxLDL-induced VSMCs. Interestin-gly, when VSMCs were treated with both oxLDL and adiponectin there was an additional effect on osteonectin expression (3.24 ± 0.39- and 24.93 ± 2.15-fold after 24 and 48 hours, respectively) (P=0.001) (Figures 1A, 2A).

The results were confirmed by western blot analysis at protein levels. oxLDL alone and together with adiponectin increased the osteonectin protein level (Figures 1B, 2B). Beta actin (42 kDa) was used as an internal control to standardize the protein loading in western blotting experiments.

## Discussion

Blood osteonectin concentration correlated significantly with atherosclerosis, stenosis and calcinosis of coronary arteries (20). In the present study, oxLDL increased osteonectin expression in VSMCs. Other studies have shown over expression of osteopontin and alkaline phosphatase in response to oxidative stress (21).

Adiponectin is involved in atherosclerosis (22, 23) and the anti-inflammatory effects of this adipocytokine have been already demonstrated. There are few reports on the preservative effects of adiponectin in arterial wall calcification (24, 25) but the adverse effects of adiponectin have been reported (26, 27). In a study, the relationship between adiponectin and coronary heart disease in old adults was investigated and the direct correla-tion between high adiponectin and the risk of coronary heart disease was reported (28).

Our results showed that adiponectin intensi-fied the oxLDL-induced increase in osteonectin level.

Luo et al. reported that adiponectin increased osteoblast cells proliferation and could raise alkaline phosphatase, osteocalcin and collagen type 2 proteins (29). Therefore, the increase in osteonectin expression, one of the secreted proteins by osteoblast in VSMCs, is not surprising.

As seen in this experimental study, if a synergistic effect exists between adiponectin and oxLDL, adiponectin may play a role in the intensi-fication of vascular calcification.

An experimental study reported that adiponectin would stop alkaline phosphatase activity, osteonectin secretion, Runx2 protein expression, and mineralization in VMSCs (30). It means that adiponectin may act as a factor influencing the decrease of proteins involved in calcification. The adiponectin density used in that study was 30 µg/ mL and its effect lasted for approximately 20 days. That study was also performed *in vivo*, which seems to be the reason for the findings’ inconsistency. There is no clear and definite data about inhibitory or activating effects of osteonectin on vascular calcification. If osteonectin reduces vascular calcification, adiponectin will have preservative effect against calcification and if osteonectin exacerbates calcification process, adiponectin can enhance calcification and therefore is a risk factor, besides the oxLDL, for calcification. In conclusion, regarding osteonectin role as an activating factor of calcification, oxLDL could act as a factor of increasing osteonectin gene expression in VSMCs, with adiponectin intensifing this process.
